# The Influence of Climacteric Symptoms on Women’s Lives and Activities

**DOI:** 10.3390/ijerph120403835

**Published:** 2015-04-03

**Authors:** Agnieszka Bień, Ewa Rzońca, Grażyna Iwanowicz-Palus, Małgorzata Pańczyk-Szeptuch

**Affiliations:** 1 Independent Obstetric Skills Unit, Faculty of Health Sciences, Medical University of Lublin, 4 Staszica St., 20-081 Lublin, Poland; E-Mails: eva.rzonca@gmail.com (E.Rz.); spupalus@gmail.com (G.I.-P.); 2 Department of Gynecology, Obstetrics and Pathology of Pregnancy, John Paul II Independent Public Regional Hospital, 10 Aleje Jana Pawła II St., 22-400 Zamość, Lublin, Poland; E-Mail: malgosiapanczyk@wp.pl

**Keywords:** climacteric, quality of life, life experiences, women

## Abstract

In the present study, we performed an analysis of the influence of climacteric symptoms on women’s lives and activities, *i.e.* their quality of life (QoL). The study was performed between October 2011 and February 2012. It included 148 women aged 44–62. The study used a diagnostic survey with questionnaires. The research instrument was the Blatt-Kupperman index. The respondents were asked to complete a questionnaire on socio-demographic data, quality of life, and the influence of climacteric symptoms on life and its various aspects. The respondents experiencing moderate or severe climacteric symptoms also had stronger feelings of failure (*p* = 0.005), feeling that opportunities are still available (*p* = 0.002), of losing their youth and beauty (*p* < 0.0001), compared to those who had slight or no symptoms. The intensity of climacteric symptoms significantly affects women’s lives and activities, *i.e.*, their QoL. The reported intensity of climacteric symptoms is influenced by the respondents’ education, residence, marital status and professional activity. The more severe the climacteric symptoms, the lower the women’s quality of life, as evidenced for example by the feeling of failure and of losing one’s youth, beauty and opportunities.

## 1. Introduction

The climacteric is a transitional period between the reproductive age and old age that spans several years before the last menstrual period and several years after that [1]. Taking into account the life expectancy of women in developed countries, it has been shown that the post-menopausal period constitutes a third of a woman’s lifetime. As regards menopause, it is a natural process in the life cycle of women, whereby menstruation becomes absent due to a cessation of the hormonal activity of the ovaries [1,2,3].

The most commonly reported menopausal symptoms include hormone withdrawal symptoms (e.g., hot flushes, night sweats, sleep disorders), as well as urogynecological and cardiovascular symptoms and complaints, osteoporosis and atrophies [2,4,5,6,7,8,9,10]. Women also report a feeling of emptiness resulting from the loss of their youth, attractiveness or social and professional roles, which affects their psycho-social functioning [2,4,6,10]. A woman’s experience of menopause is a product of interacting biological, psychological and social factors. These factors and symptoms affect a woman’s life and activities, *i.e.*, her quality of life [4,6,8,10,11,12].

Quality of life (QoL) is a broad, multidimensional concept, including physiological, psychological and social factors, as well as the subjective experience of both the negative and positive aspects of an individual’s life [13]. It is a subjective measure, reflecting the woman’s view of her health, rather than an objective evaluation of its components. QoL is an important public health issue. The quality of life of perimenopausal women affects the roles they perform, and thus, whole families and communities [13,14].

The authors of the present paper attempted an analysis of the climacteric symptoms’ influence on women’s lives and activities, *i.e.*, their QoL. The authors attempted an analysis of the influence of climacteric symptoms on women’s lives and activities, *i.e.*, their quality of life (QoL). We assessed the influence of climacteric symptoms and of their intensity on the subjective perception of the passing of time and of selected aspects of daily life.

## 2. Materials and Methods

### 2.1. Subjects

The study was performed between October 2011 and February 2012. It included 148 women aged 44–62, patients of the obstetrics-gynecology ward and clinic of the Pope John Paul II Independent Public Regional Hospital in Zamość. The women included in the study had been admitted for routine gynecological examinations or for one-day surgery. Inclusion criteria were: the occurrence of climacteric symptoms and consent to participation in the study. Exclusion criteria were: the inability to understand the questionnaire, and diagnosed gynecologic tumors. The study was performed in compliance with the Declaration of Helsinki, and had been approved by the Ethical Committee of the Polish Midwives’ Association (permission No. VI/EC/2011/PMA). All the patients were informed that their participation was voluntary and anonymous, and that all results would be used only for research purposes.

### 2.2. Assessments

The research instruments were: the Blatt-Kupperman Index [15]. The index was a combination of self-report and physician ratings. The index included 11 symptoms (vasomotor, paraesthesia, insomnia, nervousness, melancholia, vertigo, weakness (fatigue), arthralgia and myalgia, headaches, palpitations and formication) rated on a 4-point scale. To calculate the Blatt-Kupperman Menopausal Index, the symptoms were weighted as follows: hot flushes (×4), paraesthesia (×2), insomnia (×2), nervousness (×2), and all other symptoms (×1). The highest potential score is thus 51. The scores obtained were interpreted as follows: 0–16 points—no menopausal symptoms, 17–25 points—mild symptoms, 26–30 points—moderate symptoms, and more than 30 points—severe symptoms.

The respondents were asked to complete a questionnaire on socio-demographic data (age, education, residence, marital status, professional activity, and subjective health), quality of life, and the influence of climacteric symptoms on life and its various aspects. The respondents’ QoL was scored based on their answers to questions relating to the influence of menopause on: sleep, the perception of one’s appearance, the scope of family and household duties performed by the partner and children, future plans, emotional state, and sexual relationship with the husband/partner. The answers were scored 1–5 (1—strong negative influence, 5—no or positive influence). The maximum score was 100 points. Climacteric symptoms’ influence on life was rated using a 1–5 scale (1—strongly agree, 2—agree, 3—undecided, 4—disagree, 5—strongly disagree). Climacteric symptoms’ influence on selected aspects life was rated using a 5-item Likert scale (1—strongly agree, 2—agree, 3—undecided, 4—disagree, 5—strongly disagree).

### 2.3. Statistical Analyses

Statistical analysis was performed for all results. Database and statistical data were analyzed using computer software STATISTICA 12.0 (StatSoft, Cracow, Poland). Quantitative parameters were presented using means, median values, quartiles and standard deviations; qualitative ones were presented as numbers and percentages. For quantitative parameters, normality was tested using the W Shapiro-Wilk test. For comparing two independent groups, the Mann-Whitney test was used. For comparing more than two groups, the Kruskal-Wallis test was used. For finding differences between the groups in terms of qualitative variables, the Chi-squared test was used. Correlations between variables were measured using Spearman’s R. Statistical significance was set at *p* < 0.05.

## 3. Results

Among the 148 women included in the study, most were aged 50–55 (41.22%), high-school educated (57.43%), living in county capitals (60.81%), married (70.27%), professionally active (74.32%), and perceived their health as good or very good (82.43%)—Table 1.

**Table 1:** Participants’ characteristics.

Socio-Demographic Data		*n*	*%*
Age (*years old*)	Under 50 y/o	50	33.78
	50–55	61	41.22
	Over 55 y/o	37	25.00
Education	Primary/vocational	25	16.89
	High school	85	57.43
	College/university	38	25.68
Residence	Urban—province capital	12	8.11
	Urban—county capital	90	60.81
	Urban—other	11	7.43
	Rural	35	23.65
Marital status	Single	44	29.72
	Married	104	70.27
Professional activity	Working professionally	110	74.32
	Not working	38	25.67
Subjective health	Poor	13	8.78
	Good/very good	122	82.43
	Variable	13	8.78

The study showed that 33.1% of the women experienced moderate to severe menopausal symptoms, and 30.41% experienced slight symptoms (Table 2). The most distressing menopausal symptoms, according to the women, were: hot flushes (38.51%), irritability (31.76%) and insomnia (27.70%), with fewer respondents naming joint pain and headaches (23.65%), excess perspiration (20.95%) and mood swings (20.27%).

Statistical analysis showed that moderate to severe menopausal symptoms were experienced more often by respondents who were unemployed (*p* < 0.05), single (*p* < 0.05), lived in small towns or villages (*p* < 0.001), and had had primary or vocational education (*p* < 0.05). No correlation between climacteric symptom intensity and age was found (*p* > 0.05) (Table 2).

**Table 2:** Reported intensity of climacteric symptoms broken down by socio-demographic data (n = 148).

**Socio-Demographic Data**	**Intensity of Climacteric Symptoms**					
	**No symptoms**		**Slight**		**Moderate/Severe**	
	*n*	%	*n*	%	*n*	%
	54	36.48	45	30.41	49	33.10
**Age (*years old*)**						
Under 50 y/o	15	30.00	19	38.00	16	32.00
50–55 y/o	20	29.51	18	29.51	23	37.70
Over 55 y/o	19	21.62	8	21.62	10	27.03
Statistical analysis: Chi^2^ = 5.770794; *p* > 0.05						
**Education**						
Primary/vocational	7	28.00	3	12.00	15	60.00
High school	32	37.65	29	34.12	24	28.24
College/university	15	39.47	13	34.21	10	26.32
Statistical analysis: Chi^2^ = 10.56916; *p* < 0.05						
**Residence**						
Urban—province/county capital	41	40.20	38	37.25	23	22.55
Urban—small town or rural	13	28.26	7	15.22	26	56.52
Statistical analysis: Chi^2^ = 17.3529; *p* < 0.001						
**Marital Status**						
Single	14	31.82	9	20.45	21	47.73
Married	40	38.46	36	34.62	28	26.92
Statistical analysis: Chi^2^ = 6.455115; *p* < 0.05						
**Professional Activity**						
Working professionally	44	40.00	36	32.73	30	27.27
Not working	10	26.32	9	23.68	19	50.00
Statistical analysis: Chi^2^ = 6.615438; *p* < 0.05						

Table 3 shows quality of life scores in relation to the intensity of climacteric symptoms. The mean QoL score was 85.69 ± 9.15 points (Me = 88.00 points). Cronbach’s α was 0.65. The respondents experiencing moderate to severe climacteric symptoms reported a significantly lower quality of life than those with slight or no symptoms (*p* < 0.0001). Statistical analysis showed that a lower quality of life was reported by respondents who had had primary or vocational education (*p* = 0.005), lived in towns or villages (*p* = 0.007), were not professionally active (*p* = 0.05), and perceived their health as poor (*p* < 0.0001) (Table 3).

**Table 3:** Reported quality of life broken down by symptom intensity and socio-demographic data.

QoL and Intensity of Climacteric Symptoms	Mean	Standard Deviation	Lower Quartile	Median	Upper Quartile
No symptoms	91.74	5.41	89.00	93.00	96.00
Slight	85.82	7.03	82.00	86.00	91.00
Moderate/severe	79.00	9.66	72.00	80.00	88.00
Statistical analysis: H = 50.84; *p* < 0.0001					
**QoL and Education**					
Primary/vocational	79.88	9.95	73.00	78.00	88.00
High school	87.06	8.39	82.00	88.00	93.00
College/university	86.58	9.07	84.00	88.00	93.00
Statistical analysis: H = 10.48; *p* = 0.005					
**QoL and Residence**					
Urban—province/county capital	87.18	8.39	83.00	88.00	93.00
Urban—small town or rural	82.50	10.08	76.00	83.50	90.00
Statistical analysis: Z = 2.72; *p* = 0.007					
**QoL and Professional Activity**					
Working	86.63	8.84	82.00	88.00	93.00
Not working	83.11	9.73	75.00	85.00	91.00
Statistical analysis: Z = 1.99; *p* = 0.05					
**QoL and Subjective Health**					
Poor	75.62	7.73	72.00	77.00	80.00
Good/very good	88.11	7.36	85.00	89.00	93.00
Variable	73.46	9.67	68.00	72.00	80.00
Statistical analysis: H = 21.08; *p* < 0.0001					

The study showed that climacteric symptoms most strongly influenced the respondents' feelings of losing their youth, beauty and opportunities, and slightly less strongly influenced the feeling that opportunities are still available. The respondents experiencing moderate or severe climacteric symptoms also had stronger feelings of failure (*p* = 0.005), feeling that opportunities are still available (*p* = 0.002), of losing their youth and beauty (*p* < 0.0001), compared to those who had slight or no symptoms. The respondents with no such symptoms had a stronger feeling that opportunities were still available (*p* = 0.02) and stronger feelings of success (*p* = 0.09) (Table 4).

**Table 4:** Assessing the climacteric symptoms’ influence on life.

Type of Experience	Climacteric Symptoms’ Influence on Life		Climacteric Symptoms’ Influence on Life in Relation to the Intensity of Symptoms						
			No Symptoms		Slight		Moderate/Severe		Statistical Analysis
	Mean	SD	Mean	SD	Mean	SD	Mean	SD	
Feeling of success	3.64	1.05	3.44	0.96	3.62	1.03	3.86	1.14	H = 4.93
									*p* = 0.09
Feeling of failure	3.42	1.20	3.85	0.98	3.33	1.17	3.02	1.31	H=10.79
									*p* = 0.005
Feeling that opportunities are still available	3.35	1.10	3.20	1.05	3.16	1.02	3.69	1.16	H = 7.75
									*p* = 0.02
Feeling that opportunities have been lost	3.26	1.28	3.87	0.95	3.18	1.35	2.65	1.23	H = 22.90
									*p* < 0.0001
Feeling that youth and beauty pass	2.39	1.29	2.98	1.27	2.11	1.15	1.98	1.22	F = 10.36
									*p* < 0.001

Compared to the respondents with slight or no climacteric symptoms, those who experienced moderate or severe symptoms significantly more often felt this affected their relationships with their partners (*p* < 0.0001), their sexual lives (*p* < 0.00001), and their appearance (*p* < 0.0001); it also limited them in their daily activities (*p* < 0.000001), made them seek medical care more often (*p* < 0.000001), reduced their motivation (*p* < 0.000001), and necessitated the use of analgesics (*p* < 0.000001) (Table 5).

**Table 5:** Assessment of climacteric symptoms’ influence on various aspects of life.

Type of Experience	Climacteric Symptoms’ Influence on Various Aspects of Life – Domain Affected		Assessment of the Climacteric Symptoms’ Influence on Life in Relation to the Intensity of Symptoms						
			No Symptoms		Slight		Moderate/Severe		Statistical Analysis
	Mean	SD	Mean	SD	Mean	SD	Mean	SD	
Worse relationship with the partner	3.68	1.13	4.15	0.94	3.64	1.13	3.20	1.12	H = 18.84
									*p* = 0.0001
Sexual relations becoming unpleasant	3.26	1.22	3.81	0.99	3.22	1.18	2.67	1.23	H = 21.87
									*p* < 0.0001
Changes in appearance resulting in feeling unattractive	3.09	1.33	3.70	1.13	2.82	1.27	2.65	1.38	H = 18.40
									*p* = 0.0001
Limitations in daily activities	3.53	1.23	4.26	0.76	3.53	1.18	2.71	1.19	H = 40.12
									*p* < 0.0001
Lack of motivation and vital energy	3.19	1.34	3.93	1.04	2.96	1.40	2.59	1.22	H = 27.33
									*p* < 0.0001
More frequent doctor visits Family doctor, specialists	3.16	1.38	3.80	1.14	3.27	1.34	2.35	1.28	H = 28.13
									*p* < 0.0001
Pain (back pain, joint pain, headaches) necessitating the use of analgesics	2.80	1.40	3.56	1.33	2.82	1.32	1.94	1.03	H = 35.66
									*p* < 0.0001

Statistical analysis showed a significant correlation between QoL and experiences such as the feeling of success (R = −0.19). The higher the QoL, the stronger the feeling of success. A higher QoL also reduces the feelings of failure (R = 0.43), lost opportunities (R = 0.35) and passing beauty (R = 0.37) (Table 6).

**Table 6:** Correlations between the influence of climacteric symptoms on life, and the quality of life.

Type of Experience	Statistical Analysis	
	*R*	*p*
Feeling of success	−0.19	0.02
Feeling of failure	0.43	<0.000001
Feeling that opportunities are still available	−0.12	0.15
Feeling that opportunities have been lost	0.35	0.00001
Feeling that youth and beauty pass	0.37	0.000003

Statistical analysis showed significant correlations between QoL and the influence of climacteric symptoms on specific aspects of life. The correlations were rated at between 0.27 and 0.55. A strong influence of climacteric symptoms on specific aspects of life was found to be correlated with a lower quality of life (Table 7).

**Table 7:** Correlations between QoL and the strength of climacteric symptoms’ influence on various aspects of life.

Aspects of Life	Statistical Analysis	
	*R*	*p*
Worse relationship with the partner	0.51	<0.000001
Sexual relations becoming unpleasant	0.55	<0.000001
Changes in appearance resulting in feeling unattractive	0.40	<0.000001
Limitations in daily activities	0.49	<0.000001
Lack of motivation and vital energy	0.45	<0.000001
More frequent doctor visits family doctor, specialists	0.47	<0.000001
Pain (back pain, joint pain, headaches) necessitating the use of analgesics	−0.27	0.0009

## 4. Discussion

Although the climacteric is a natural transition occurring in a woman’s life, the literature produced worldwide reports many problems experienced at that time [1,2,4,8,9,10,13,14]. The present paper attempted an analysis of the influence of climacteric symptoms on the quality of life and on various aspects of the lives of women living in Zamość, Lublin, eastern Poland. We assessed the influence of the climacteric symptoms and of their intensity on the subjective perception of the passing of time and of selected aspects of daily life, including the relationship with the partner, attractiveness, daily activity, and the need for medical care. The study employed the Blatt-Kupperman index to assess the intensity of climacteric symptoms in the studied group. The instrument’s reliability was high (Cronbach’s α = 0.86). The authors’ own analysis shows that severe climacteric symptoms and the low quality of life experienced by the study participants are correlated with primary or vocational education, the lack of professional activity, and rural residence. The results obtained by the authors of this study reflect those obtained by Le *et al.* [16] in Sweden, also indicating that higher education levels of women were significantly correlated with fewer complaints related to climacteric symptoms. This is corroborated by Wieder-Hulsza *et al.* [17], whose study showed a statistically significant correlation between the education level and professional activity of post-menopausal women, and their QoL. In their longitudinal study, Moilanen and colleagues [18] found that an improvement in global quality of life is correlated with stable or increased physical activity, stable weight and high education. Bould and Brown [4] reported that women with a high level of emotional intelligence have a more positive attitude towards menopause, experience less stress, psychological tension and menopausal symptoms, and have a better perception of their physical health. Zagozdzon *et al.* [13], Żołnierczuk-Kieliszek *et al.* [14] and Iwanowicz-Palus *et al.* [10] emphasize that residence affects the QoL of women during the climacteric, and specifically that rural residence means a lower quality of life, both in physical and psychological terms.

The subgroup reporting the lowest quality of life included women who had had primary education, lived in towns or villages, were not active professionally and perceived their health as poor. In Poland, these are mainly women having a low socio-economic status. The study was performed in a time of economic stability in the country. However, according to the Polish Central Statistical Office, the quality of life in Poland, especially in rural areas, remains unsatisfactory [19].

Although the authors’ own study showed no correlation between climacteric symptom intensity, quality of life, and age, other studies prove that the post-menopause quality of life depends on many factors, including age. Middle age is a risk factor for a lower quality of life, which is related to poorer subjective physical health and more frequent health complaints [12,14,20,21].

A lower QoL is correlated with more intense climacteric symptoms, which is confirmed by the authors' own study, as well as by Wieder-Hulsza *et al.* [17] and Poomalar and Arounassalame [22]. The study did not, however, investigate correlations between the time of climacteric symptom onset and the intensity or perception of symptoms. Therefore, the possible link between the intensity of the symptoms and their duration cannot be denied. This might indirectly influence the studied women’s reported quality of life.

Health, family and work are important to midlife women [2]. The results of the authors’ study showed that better-educated and professionally active women enjoyed a higher quality of life. Results obtained by Williams *et al.* [23] in a representative sample of 2703 American women aged 40–65, having undergone natural or surgical menopause, also showed a correlation between better education and a higher quality of life. The authors showed that women with a higher level of education enjoyed a significantly higher QoL than those with a lower level of education. The authors also confirmed that professionally active women had a higher quality of life than their professionally inactive counterparts.

Professional activity and rural residence are important factors in perimenopausal women’s overall wellbeing; most women living in rural areas do not take proper care of their health [10]. Furthermore, work-related stress and difficult working conditions contribute to the severity of climacteric symptoms [6]. In the present study, most respondents were professionally active. It should be noted that in Poland, employment does not guarantee a higher quality of life. However, the authors’ own study showed that professionally active respondents experienced fewer hormone withdrawal symptoms and enjoyed a better quality of life. Zagozdzon and colleagues [13] report that factors lowering the health-related QoL of women living in rural areas include: being retired or receiving disability pension, experiencing chronic illness symptoms, and receiving specialist medical care.

An analysis of climacteric symptoms’ influence on various aspects of life showed that the influence was the strongest in terms of: the use of analgesics due to various types of pain (back pain, joint pain, headaches); changes in appearance affecting the woman’s attractiveness; the need to seek medical care more often; and lowered motivation and energy. Szpak *et al.* [1], in their study on midlife women’s relationship with their partners, reported that satisfaction with the affective aspect of the relationship differs by sex. Male participants declared they were satisfied or very satisfied with their relationship. As regards female participants, their affective satisfaction was moderate. The same applies to the subjective quality of sexual relations.

The sexual behaviors of middle-aged women are affected by many factors, including their general health, relationship with the partner, and overall life situation [24]. The authors’ own analysis shows that respondents experiencing moderate to severe climacteric symptoms also reported a more significant decrease in the quality of their relationship or sex life, and stronger feelings of waning beauty and attractiveness. Ornat and colleagues [25] reported that low sexual function or a low satisfaction with sex life in middle-aged women is correlated with age, menopausal symptoms, and a low satisfaction with life. Notably, post-menopausal women often feel less physically attractive [2]. As reported by Szpak *et al.* [1] 75.86% of male respondents still consider their partners attractive after menopause.

The study showed that a higher intensity of climacteric symptoms adversely affected women’s lives and activities, as well as their subjective perception of the passing of time and of selected aspects of daily life, including the relationship with the partner, attractiveness, daily activity, and the need for medical care. Strong influence of climacteric symptoms on specific aspects of life was found to be correlated with a lower quality of life. Intense climacteric symptoms adversely affect women’s daily life and their motivation. It would be advisable to educate all women on various methods and options for alleviating climacteric symptoms, which might positively influence their daily lives and their ability to cope in difficult situations. Such efforts should be intensified especially in rural environments. Such education on the climacteric and on ways of managing it must include the women’s families as well as the women themselves. An emphasis on the role of the family in coping with the situation could improve family members’ understanding of the women’s behavior.

## 5. Conclusions

The reported intensity of climacteric symptoms is influenced by the respondent’s education, residence, marital status and professional activity.

The intensity of climacteric symptoms significantly affects women's lives and activities, *i.e.*, their quality of life.

The more severe the climacteric symptoms, the lower the women’s quality of life, as evidenced, among other experiences, by feelings of failure and of losing one’s youth, beauty and opportunities.

Increased intensity of climacteric symptoms adversely affects various aspects of women’s daily lives, such as their sex life, relationship with the partner or their perceived attractiveness; it also limits them in daily activities, makes them seek medical care more often, reduces their motivation and necessitates the use of analgesics.
